# eHealth and the hearing aid adult patient journey: a state-of-the-art review

**DOI:** 10.1186/s12938-018-0531-3

**Published:** 2018-07-31

**Authors:** Alessia Paglialonga, Annette Cleveland Nielsen, Elisabeth Ingo, Caitlin Barr, Ariane Laplante-Lévesque

**Affiliations:** 1 National Research Council of Italy (CNR), Institute of Electronics, Computer and Telecommunication Engineering (IEIIT), Piazza Leonardo da Vinci 32, 20133 Milan, Italy; 2 Eriksholm Research Centre, Oticon A/S, Snekkersten, Denmark; 3 Department of Behavioural Sciences and Learning, Linköping University, Linköping, Sweden; 4 Department of Audiology and Speech Pathology, University of Melbourne, Melbourne, Australia; 5 Oticon Medical, Vallauris, France

**Keywords:** eHealth, Hearing aids, Hearing loss, Hearing rehabilitation, mHealth, State-of-the-art review

## Abstract

The number and variety of eHealth services for adults and older adults who use hearing aids (HAs) are growing rapidly. This area holds promise to increase cost-efficiency, enable better access to care, and improve patient outcomes and satisfaction. Despite the increasing interest in this field, an up-to-date picture of recent research in the area of eHealth for adults with HAs is lacking. In this state-of-the-art review we assessed the literature from the past decade about eHealth use in the HA adult patient journey. Systematic searches were conducted in CINAHL, PubMed, Scopus, and Web of Science. A total of 34 peer-reviewed empirical records were identified from the searches and from the reference lists of searched records. Records were characterized based on: eHealth platform (i.e.: *offline*, *Internet*-*based*, or *mobile-based*), service [i.e.: *education and information*, *screening and assessment*, *hearing rehabilitation*, or *general (tele*-*audiology)*], and phase of the patient journey (i.e.: *pre*-*fitting*, *fitting*, or *post*-*fitting*). The review highlighted a growing interest in the field, as revealed by an increasing trend over the search period, from 2 records in 2009–2010 up to 17 records in 2015–2016. *Internet*-*based* platforms were the most frequently used (present in more than half of the included records), with a stable trend in the period. About one-third of the records introduced services over *offline* platforms, whereas *mobile*-*based* platforms were used only in 6 out of 34 records, suggesting that the clinical uptake of mobile services is still limited compared to more mature *offline* and *Internet*-*based* platforms. Most of the eHealth services observed were related to the areas of *education and information* (42.5%) and *hearing rehabilitation* (40.4%), whereas 10.7% were related to *screening and assessment,* and 6.4% to general tele-audiology services. Many services covered different phases of the patient journey, especially the *fitting* and *post*-*fitting* phases. Overall, this review showed that the field of eHealth in the context of HA rehabilitation in adults has grown in the recent past. Research is still needed to increase the uptake and efficacy of eHealth in clinical practice, especially in terms of technology developments, technical and clinical validation, and optimization of strategies for service delivery.

**Electronic supplementary material:**

The online version of this article (10.1186/s12938-018-0531-3) contains supplementary material, which is available to authorized users.

## Background

We are witnessing a rapid growth in the area of eHealth for hearing healthcare (HHC) and this opens several novel, promising opportunities for hearing professionals as well as for their patients. The number and variety of eHealth solutions available for the different target user groups and their penetration into clinical practice are growing [1–5]. There is ample debate and interest about the benefits and challenges of eHealth in HHC: for example, whilst many hope for greater cost efficiency and access to care for people living in underserved communities, too few examples of sustainable eHealth-enabled services currently exist [6–11].

Several definitions of eHealth have been introduced throughout the years (see, e.g., [12–15]). In this manuscript, we follow the World Health Organization (WHO) definitions [13, 14] that describe eHealth as broad in scope and capture its several aspects. Specifically, the “Resolution on eHealth” adopted by the WHO in 2005 defined electronic health (referred to as eHealth) as the *“use of information and communication technologies (ICT) locally and at a distance”* in support of health and health-related fields including, e.g., health care services, surveillance, and health promotion [13]. This definition highlighted that eHealth may include both local and remote services, therefore we did not limit our analysis to remote services only and we also included computer-based services. The 2016 WHO definition introduced new perspectives on the scope and services of eHealth [14]. This document described eHealth as “*the use of electronic means to deliver information, resources and services related to health. It covers many domains, including electronic health records, mobile health, and health analytics, among others”.* eHealth can put information in the right place at the right time, providing more services to a wider population and in a personalized manner [14]. This newer definition highlighted the latest aspects of the digital health revolution such as health analytics, computational tools, and mobile health and as such it was used in this manuscript to assess the most recent solutions and capture the newest trends in eHealth in the area of hearing rehabilitation.

Throughout the years, deep changes have occurred in the way in which eHealth can be delivered, not only in terms of offered services and functions, but also in terms of platforms for service delivery. Examples of platforms in eHealth range from tele-health based on digital video disks (DVDs), personal computer (PC)-based applications, or telephone services (referred to as *offline* platforms in this study) to more recent solutions delivered over the Internet (referred to as *Internet*-*based* platforms) or by means of mobile and wearable devices (referred to as *mobile-based* platforms). In particular, the mobile health (mHealth) branch is growing rapidly due to ubiquitous use of personal mobile devices such as smartphones, phablets, and tablets, and due to the related increase in popularity of mobile health applications (apps) [16–18]. Noticeably, recent market estimates reported that about 325,000 health apps were available on the leading app stores in 2017 [19]. Also in audiology and related fields apps are abundant [5, 6], but few are used [11].

In the field of audiology, Cherry and Rubinstein suggested the use of eHealth for patient follow-up as early as in 1994 [20], introducing remote follow-up by telephone following face-to-face fittings of hearing aids (HAs). Use of eHealth has grown since then and, to date, several solutions are available for various hearing-related conditions (e.g., hearing loss (HL), tinnitus, balance problems) and for different target user groups (e.g., children with cochlear implants, parents of children with HL, adults fitted with HAs or implantable devices, older adults, patients suffering from tinnitus) [6, 7, 10]. Recently, the ecosystem of eHealth solutions has grown further and includes interesting developments in the field of mobile apps for patients and their families, for professionals, or for the general population [3, 5, 6, 21]. In general, eHealth promises to pave the way to more effective hearing healthcare for adults and might be helpful to leverage patient outcomes and satisfaction with its several applications and potential benefits, e.g. in data collection, teleconsultation, remote monitoring, remote fitting, or delivery of educational programs for patients (e.g., [7, 10, 22, 23]). Use of eHealth can be effective in terms of cost, resources, and time and enables personalized approaches to hearing rehabilitation, for example in terms of content, depth of detail, and interfaces. eHealth based rehabilitation programs have the advantage over face-to-face encounters of being more quickly interactive and can provide immediate and useful feedback to the user such as test results or advice [10]. Moreover, remote provision of hearing services through eHealth can support the advancement of public health policies on hearing rehabilitation, especially in remote areas and can, in general, it can bring technology closer to patients, increase motivation, and promote behavior change [23]. These benefits can be enhanced by patient segmentation strategies and customization of services, in a way that is intended to be patient-centered to meet the individual needs [24].

A very large—and growing—target group for eHealth in HHC is the population of adults and older adults with HL. In this group, HL can be age-related, noise-induced, medication-induced, or due to other possible causes. In fact, the WHO reported HL as the first among the 20 leading causes of moderate-to-severe disability worldwide, with 278 million people in the world estimated to suffer from moderate-to-severe loss in both ears [25, 26]. In the older age groups, in particular, hearing impairment is a very common chronic condition. It is estimated that 46% of persons over the age of 60 years, and as many as 83% over 70 years, experience some degree of HL, with an incidence of about 1 in 25 older adults each year [27]. In 2008, the WHO estimated the number of people aged 60 years and over that experienced moderate-to-severe HL: about 18.5 million in high-income countries and about 43.9 million in low- and middle-income countries [25]. Hearing impairment can bring along considerable burden on the individual in terms of speech communication, social participation, personal well-being, productivity on the workforce, quality of life, and cognitive decline [28–30]. The burden on the population is likely to increase further in the next years due to aging and delayed retirement, thus creating more economic pressure on the healthcare systems [31, 32]. In 2017, the WHO estimated the annual global cost of unaddressed HL to reach 750 billion international dollars: this includes health sector costs (excluding the cost of hearing devices), costs of educational support, loss of productivity, and societal costs [33].

For adults and older adults with HL, the most common rehabilitation option is HA fitting [34]. HAs can be efficacious in older adults, especially when delivered using audiology best practices [35]. However, HA adoption and effective use is related to many factors and it may happen that people fitted with HAs either fail to use them or do not gain optimal benefit—especially the older people [36–39]. In addition, many people who could benefit from HAs typically delay seeking help, often for more than 10 years so there is a large unmet need [40]. Efforts have been made throughout the years to increase the number of people who receive a diagnosis and effective rehabilitation so to try to limit the burden of untreated HL in growing population of adults and older adults. Examples include developments in technology [34, 41, 42], improvements in intervention strategies [43], development of methods for hearing screening and early rehabilitation [44–48] and, in the recent years, the introduction of eHealth into the diagnosis and care process. However, despite the growing interest in this field, an up-to-date picture of the eHealth solutions introduced recently in the context of HA rehabilitation in adults is lacking.

The aim of this study was to review the recent literature about eHealth use in all clinical activities supporting the HA adult patient journey. This state-of-the-art review aims to provide a picture of the recent evolution of eHealth in this area. Such an overview of the current state of knowledge can be useful to audiologists to help them understand the recent trends in the field and the technology available for their clinical practice, the associated benefits as well as possible barriers to implementation. It can also be useful to researchers in this area to understand the current research needs in this field as well as opportunities for further developments.

## Methods

### Search strategy

As this state-of-the-art review encompasses, in principle, both quantitative and qualitative studies the search question was formulated by using the broader PICo approach for qualitative studies (Population, Interest, and Context). Specifically, we focused on adults with HL (Population) and we addressed recent research in the area of clinical use of eHealth (Interest) for hearing rehabilitation by use of HAs (Context).

Systematic searches were conducted in four databases: CINAHL, PubMed, Scopus, and Web of Science. The main search string included terms related to eHealth, computers, fitting, consulting, HAs, HL, adults, and excluded terms related to infants and children, as well as cochlear implants: (“hearing aid” OR “sensory aid” OR “hearing instrument” OR “hearing loss” OR “hearing-related” OR “hearing healthcare”) AND (Internet OR eHealth OR mhealth OR mobile OR remote* OR “tele-audiology” OR online OR “face to face” OR “tele*health” OR tele-hearing OR “telemedicine” OR telepractice OR Comput* OR “PC” OR “Computer*assist*” OR “Computer*aided” OR “Computer*based” OR “consulting” OR “fitt*”) NOT (children OR babies OR cochlear implant* OR implant OR “FM” OR “noise” OR “HIV” OR “CLS” OR “ELS” OR “visual*” OR “otitis media” OR “gene” OR “sensorineural”). The main search string was modified slightly to meet the requirements of each database (see Additional file 1 for the full search strings used in the four databases). We focused on the literature from the past decade as this is the most widely used approach in state-of-the-art reviews, so to capture the recent trends in research [49]. The search included records published from 2007 to 2017 (as of May 30, 2017). Duplicates were removed before the screening. The reference lists of relevant records were also checked to identify additional relevant records. Records were screened and selected according to the following inclusion and exclusion criteria:

#### Inclusion criteria

- To fulfil the search question, records had to relate to clinical usage or clinical applications of eHealth and HA rehabilitation in adults in any phase of the patient journey (before, during, and after HAs);
- To get insight on the use and impact of eHealth, records had to be empirical and must include quantitative results on a study sample of human participants;
- According to typical literature review characteristics [49], records had to be peer-reviewed;
- To allow discussion among investigators in the transnational group, records had to be in English.

#### Exclusion criteria

- Absence of author names, title or a traceable abstract;
- Records only containing an abstract;
- Conference papers published in full elsewhere and included in full version in this review;
- Text books;
- Records exclusively related to development and validation of hearing tests and measures;
- Records exclusively on deaf and hard of hearing.

The authors independently applied the inclusion and exclusion criteria on a subset of 20 records and discussed their results to ensure that they applied the criteria homogenously. Discrepancies between the authors were resolved through discussion; reasons for excluding records were registered.

Although not mandatory in state-of-the-art reviews [49] we supplemented this review with formal quality assessment of the included records. The methodological quality was assessed by using the Downs and Black scale, which targets the domains of reporting, external validity, internal validity (including measurement bias and confounding subject selection bias), and statistical power (simplified version) [50, 51]. The scale items and scoring system is reported in detail in Additional file 2. All authors used the same software for reference management (Mendeley, Mendeley Ltd., Elsevier) and for data extraction, management, and analysis (Office Excel 2013, Microsoft Corporation).

Three investigators (AP, ACL, and ALL) analyzed the full texts for inclusion and exclusion criteria and then classified the included records as described in the following section. In case of disagreement, the results were discussed until consensus was reached.

### Analysis of records

For a descriptive assessment of eHealth in terms of platforms, services offered, and use along the phases of the adult HA patient journey, the records were coded in terms of:

- Platform for eHealth delivery, i.e.:
  i. *Offline* (e.g., DVDs, PCs, or telephone);
  ii. *Internet*-*based* (e.g., websites and web-portals, remote control of equipment, audio–video-conferencing for teleconsultation); or
  iii. *Mobile-based* (e.g., mobile and wearable technology, mobile applications).
- Service offered, as defined by four broad service areas according to [5, 6, 50]:
  i. *Education and information* (an area that may include services for improved knowledge and awareness about hearing or HL, or programs for education, learning, and training for the patients, including those delivered to complement hearing rehabilitation);
  ii. *Screening and assessment* (the area of hearing testing, that may include services for hearing screening, hearing assessment and diagnosis, or self-assessment tools);
  iii. *Hearing rehabilitation* (an area related to technology and services for hearing rehabilitation, including amplification and training); or, if the service was not specific to one or more of the above,
  iv. *General (tele*-*audiology)*.
- Phase of the patient journey, i.e.:
  i. *Pre*-*fitting* (i.e., the phase that precedes the intervention and might include stages such as awareness of HL, hearing screening, hearing assessment and diagnosis);
  ii. *Fitting* (i.e., the intervention phase, that may include sound amplification, HA fitting and adjustment, as well as behavioural and self-reported outcome measures);
  iii. *Post*-*fitting* (i.e., the phase that follows the intervention and may include, e.g., auditory rehabilitation, auditory or cognitive training, patient education and self-management).

## Results

The systematic searches in the four databases identified 338 distinct records after removing duplicates. The titles, abstracts, and reference lists of these 338 records were screened, resulting in selection of 198 full text records assessed for eligibility, including 43 records from reference lists. The full-texts of the 198 records were reviewed for inclusion and exclusion criteria, resulting in a final list of 34 empirical records related to eHealth and HAs in adults. The records showed a range of study designs: 15 were randomized controlled trials, 17 were cross-sectional/cohort-studies, and 2 were case studies. The sample size ranged from 3 (in a case study) to over 42,000 (in a retrospective cohort study). The median sample size was 59. The methodological quality scores ranged from 2 to 25 out of 28 overall. The overall scores ranged from 13 to 25 in randomised controlled trials (average: 17.7), from 2 to 21 in cross sectional/cohort studies (average: 10.2), and from 3 to 5 in case studies (average: 4.0). On average, the scores in the reporting domain were: 6.4 out of 11 overall, 7.7 in randomised controlled trials, 5.6 in cross sectional/cohort studies, and 3 in case studies. On average, external validity was low across the set (0.9 out of 3 in the whole sample) although it was relatively high in randomised controlled trials (1.3 out of 3) as more efforts were made in these studies to ensure that the study participants were representative of the population and that the experimental settings were representative of the treatment the majority of patients receive. In the domains of measurement bias and subject selection bias the average values in the 34 included records were 3.7 out of 7 and 2.6 out of 6, respectively. The scores related to measurement bias and subject selection bias were higher in randomised controlled trials (4.9 out of 6 and 4.3 out of 7, respectively), lower in cross sectional/cohort studies (3.0 out of 6 and 1.3 out of 7, respectively) and very low in the two case studies (1.0 out of 6 and 0 out of 7, respectively), due to inherent study design characteristics. Only seven studies (5 randomised controlled trials and 2 cross-sectional/cohort studies) included a calculation of statistical power. The assessment of study quality is reported in full detail in Additional file 2.

### Evolution of publications and eHealth platforms in the period 2007–2017

**Fig. 1:**
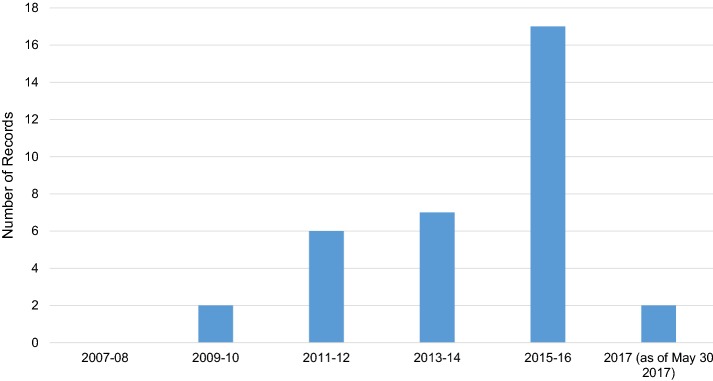
Evolution of publications in the period 2007–2017. The figure shows the number of included records as a function of the publication year

Figure 1 shows the number of included records as a function of the publication year in the period 2007–2017. No records published in 2007–2008 passed the screening. Then, in the period 2009–2016 the number of included records increased from 2 in 2009–2010 up to 17 in 2015–2016. In 2017, only 2 records were found but this relatively low number was due to a combination of factors. The search date was May 30, 2017 so it identified only the records indexed in the databases up to that date. As indexing of journal publications in databases might take up to several months, the search gave only a partial picture of the actual number of articles published in 2017 at the time of search.

**Fig. 2:**
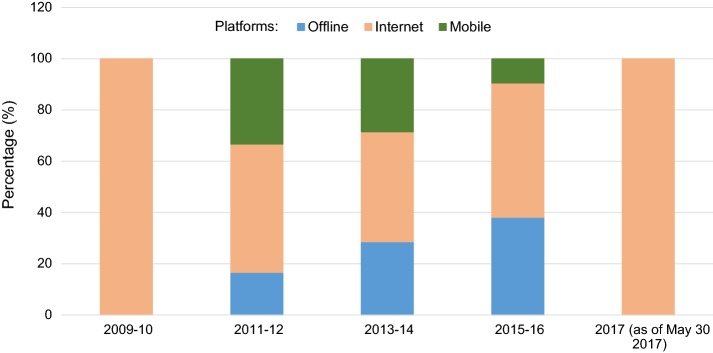
Evolution of platforms in the period 2009–2017. The figure shows the distribution (percentage values) of eHealth platforms (*offline*, *Internet*-*based*, *mobile*-*based*) across the included records as a function of the publication year

Characterization of the 34 included records in terms of the eHealth platform (*offline*, *internet*-*based*, or *mobile*-*based*) revealed that 7 records used *offline* platforms (DVD-, PC-, or telephone-based), 17 used *Internet*-*based* solutions (websites, Internet-based programs, teleconsultations), 6 presented *mobile*-*based* services (smartphone- or tablet-based systems), and 4 presented services that could be delivered over more than one platform, specifically offline and through the Internet. Figure 2 shows the distribution (percentage values) of the three platforms across the included records as a function of the publication year. Figure 2 shows that, with the exception of years 2009–2010 and 2017 (where the only two records found were related to *Internet*-*based* services only) in the period 2011–2016 the percentage of *Internet*-*based* services was almost unchanged, whereas the percentage of *offline* services increased and the percentage of *mobile*-*based* services decreased. It is worth noting that the percentage values in Fig. 2 are computed from relatively low numbers, especially in years 2011–2012 and 2013–2014, so the variability of percentage estimates is high and this may limit the strength of the observed trends. Nevertheless, in years 2015–2016 we found a relatively high number of studies, especially randomised controlled trials, which addressed DVD and PC-based services and this might suggest a trend towards increasing interest in the area of *offline* services.

### Overview of the eHealth services, platforms, and phases of the patient journey

Characterization of the 34 included records in terms of the service offered [*education and information*, *screening and assessment*, *hearing rehabilitation*, and *general (tele*-*audiology)*] is shown in Table 1. The Table shows the services and sub-services found across the included records and the number of records related to each. The service described more frequently was hearing rehabilitation (especially auditory and cognitive training), followed by education and information (especially the sub-service counselling and patient education, which is closely related to hearing rehabilitation). A lower number of records were related to screening and assessment (especially, self-assessment tools to measure, e.g., hearing handicap, fitting benefits and satisfaction) and to tele-audiology in general. Three out of 34 records included more than one service (specifically, in 2 records the hearing rehabilitation area was combined with education and information and in 1 record it was combined with screening and assessment).

**Table 1:** Overview of the eHealth services across the phases of the patient journey (N = number of records)

Services	Phases		
	Pre-fitting	Fitting	Post-fitting
(1) Education and information (N = 10)			
(1.1) HL and HA information (N = 3)	✓		✓
(1.2) Counselling and patient education (N = 10)	✓		✓
(1.3) Group discussions and forums (N = 4)	✓		✓
(2) Screening and assessment (N = 4)			
(2.1) Pure-tone audiometry (N = 1)	✓		
(2.2) Speech-audiometry (N = 2)	✓	✓	
(2.3) Self-assessment and PROM/PREM (N = 4)	✓	✓	✓
(3) Hearing rehabilitation (N = 14)			
(3.1) Sound enhancement (N = 2)		✓	
(3.2) HA control and fitting (N = 6)		✓	
(3.3) Auditory and cognitive training (N = 9)	✓		✓
(4) General (tele-audiology) (N = 3)	✓	✓	✓

*HA* hearing aid, *HL* hearing loss, *PROM* patient-reported outcome measures, *PREM* patient-reported experience measures

**Fig. 3:**
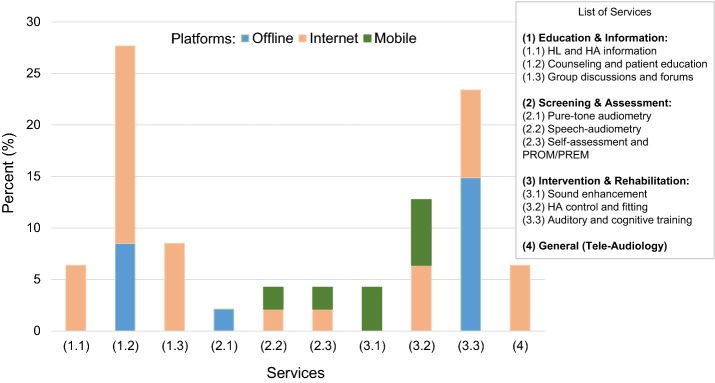
eHealth services and platforms. The figure shows the percentage values of each eHealth service and each eHealth platform across the 34 included records

Each of the eHealth services identified can, in principle, be delivered over one or more platforms (*offline*, *internet*-*based*, or *mobile*-*based*). Figure 3 summarizes the findings from the 34 included records and shows the percentage of each eHealth service and each eHealth platform across the set. *Offline* platforms were used for 3 out of 10 services, i.e.: counselling and patient education (PC- and DVD-based educational programs and telephone consultations), pure-tone audiometry (portable system), and auditory and cognitive training (PC- and DVD-based training programs). For two out of these three services (i.e., counselling and patient education and auditory and cognitive training), this review also showed records related to the *Internet*-*based* modality. In general, the *Internet*-*based* modality was used across the majority of services (i.e., in 8 out of 10). It was the only platform where we found evidence in the areas of HL and HA information (educational websites), group discussions and forums (online forums), and tele-audiology in general (remote service delivery through the Internet). For speech-audiometry and self-assessment, an equal number of records were found to be related to the *Internet*-*based* and to the *mobile*-*based* modality (Web- and smartphone-testing, respectively). The same holds for HA control and fitting (remote HA fitting by audiologists through the Internet and smartphone-based self-fitting). For sound enhancement, we found evidence only on *mobile*-*based* platforms (smartphone-based personalized hearing compensation).

eHealth services were also characterized in terms of the relevant phase(s) along the patient journey (*pre*-*fitting*, *fitting*, and *post*-*fitting*). Results are summarized in Table 1. The Table shows that some of the services identified in this review are relevant to only one phase (e.g., sound enhancement and HA control and fitting naturally belong to the *fitting* phase), whereas most of the services can, in principle, be used in one or more phases of the patient journey (e.g., services in the education and information area might, for example, be helpful to assist patients throughout their journey).

A detailed summary of the evidence found across the 34 records is reported in Table 2. Most of the records reporting services in the education and information area were related to the post-fitting phase, i.e. for informing and educating patients following HA fitting [50–61]. Evidence supporting use in the post-fitting phase was found for each of the three services in this area (Table 1). For HL and HA information, two records described Internet delivery of information about, e.g., hearing, hearing health, HAs, auditory rehabilitation, and communication strategies [50–54]. For example, Peddie and Kelly-Campbell [50] showed that adults with HL (median age 70 years; range 44–84) are likely to use the Internet as source of hearing health information. The Authors concluded that this may change the relationship between clinicians and patients who have conducted their own research in a way that use of Internet to search for HL and HA information may have an impact on the way clinicians approach their patients.

**Table 2:** Overview of the eHealth services, platforms, and phases across the included records

Service	Platform	Records	Details	Phases
(1) Education and information				
(1.1) HL and HA information	Offline	–	–	–
	Internet	Peddie and Kelly-Campbell [50]	Use of the Internet to search for hearing and hearing health information	Pre-fitting, Post-fitting
		Rothpletz et al. [62]	Use of websites to find information on HL, HAs, assistive technologies, communication strategies, and auditory training	Pre-fitting
		Thoren et al. [54]	Internet-based information for patients with HA	Post-fitting
	Mobile	–	–	–
(1.2) Counselling and patient education	Offline	Ferguson and Henshaw [55], Ferguson et al. [22, 56]	DVD- and PC-based multimedia educational support for patients (Reusable Learning Objects including videos, photos, sounds, testimonials, animations, illustrations, and subtitles)	Post-fitting
		Lundberg et al. [57]	Telephone-based consultations as a complement to a home-based educational program for HA users (10–15 min at the end of each week)	Post-fitting
	Internet	Ferguson and Henshaw [55], Ferguson et al. [22, 56]	Internet-based multimedia educational support for patients (Reusable Learning Objects including videos, photos, sounds, testimonials, animations, illustrations and subtitles)	Post-fitting
		Malmberg et al. [58]	Internet-based educational program for HA users (reading, home tasks, counselling)	Post-fitting
		Manchaiah et al. [63]	Internet-based pre-fitting counselling for persons with HL (30 days program based on the ‘patient journey’ model)	Pre-fitting
		Preminger and Rothpletz [59]	Design considerations for Internet-based educational programs (education, self-management practice)	Post-fitting
		Thoren et al. [54, 60]	Internet-based counselling and education for patients with HA (one chapter per week, 5 weeks, home tasks, weekly professional feedback)	Post-fitting
		Vitti et al. [61]	Internet-based education for patients with HA	Post-fitting
	Mobile	–	–	–
(1.3) Group discussions and forums	Offline	–	–	–
	Internet	Malmberg et al. [58]	Internet-based interaction with peers for patients with HA (unmoderated discussion forum)	Post-fitting
		Preminger and Rothpletz [59]	Design considerations for Internet-based educational programs (group discussions and peer support)	Post-fitting
		Thoren et al. [54, 60]	Internet-based interaction with peers for patients with HAs (online discussion group, one topic per week, 5 weeks)	Post-fitting
	Mobile	–	–	–
(2) Screening and assessment				
(2.1) Pure-tone audiometry	Offline	Coco et al. [64]	Community-based pure-tone audiometry with an automated portable diagnostic audiometer	Pre-fitting
	Internet	–	–	–
	Mobile	–	–	–
(2.2) Speech-audiometry	Offline	–	–	–
	Internet	Blamey et al. [65]	Internet-based speech test for hearing assessment and HA fitting (50 CVC words at 65 dBA)	Pre-fitting, Fitting
	Mobile	Sohn et al. [66]	Smartphone-based speech test for hearing assessment and compensation (4 phonemes at 20–70 dB SPL)	Pre-fitting, Fitting
(2.3) Self-assessment, PROM/PREM	Offline	–	–	–
	Internet	Thoren et al. [67]	Online administration of standardized questionnaires in patients with HL (HHIE. IOI-HA, SADL, and HADS)	Pre-fitting, Fitting
	Mobile	Galvez et al. [68]	Personal Digital Assistant for Ecological Momentary Assessment (12-items survey)	Post-fitting
(3) Hearing rehabilitation				
(3.1) Sound enhancement	Offline	–	–	–
	Internet	–	–	–
	Mobile	Amlani et al. [69]	Smartphone-based sound enhancement (user control over frequency response and gain)	Fitting
		Sohn et al. [66]	Smartphone-based sound enhancement for hearing compensation (64-channels gain and compression)	Fitting
(3.2) HA control and fitting	Offline	–	–	–
	Internet	Ferrari and Bernandez-Braga [70]	Remote probe microphone for real ear measurement in HA fitting	Fitting
		Penteado et al. [23, 71]	Remote HA fitting (video-conferencing, remote computer access, remote digital HA fitting)	Fitting
	Mobile	Aldaz et al. [72]	Smartphone-based personalized system for HA control (volume, program, microphone directionality, noise reduction)	Fitting
		Convery et al. [73]	Tablet- and Smartphone-based self-fitting of HAs (pure-tone self-testing at 3 or 3 + 3 frequencies, program control)	Fitting
		Panahi et al. [74]	Smartphone-based adaptive speech enhancement for HAs	Fitting
(3.3) Auditory and cognitive training	Offline	Chisolm et al. [75]	PC-based auditory training with the Listening and Communication Enhancement program (30 min/day, 5 days/week, 4 weeks)	Post-fitting
		Ferguson and Henshaw [55, 76], Henshaw et al. [77]	PC-based auditory training (phoneme discrimination in quiet: 15 min/day, 6 days/week for 4 weeks; phoneme discrimination in noise: 30 min/day for 7 days)	Post-fitting
		Ferguson and Henshaw [55, 76]	PC-based working memory training (verbal and visuospatial working memory and storage tasks: 35–45 min/day, 5 days/week, 5 weeks)	Post-fitting
		Olson et al. [78]	DVD-based auditory training with the Listening and Communication Enhancement program (30 min/day, 5 days/week, 4 weeks)	Post-fitting
		Rishiq et al. [79]	PC-based auditory training (audio-visual recordings of wise or witty sayings and crossword puzzle: 30 min/day, 5 days/week, 4 weeks)	Post-fitting
		Saunders et al. [80]	DVD- and PC-based auditory training with the Listening and Communication Enhancement program (30 min/day, 5 days/week, 4 weeks)	Post-fitting
	Internet	Abrams et al. [81]	Internet-based audio-visual auditory training (30 min/day, 5 days/week, 3 weeks)	Post-fitting
		Ferguson and Henshaw [55, 76]	Internet-based working memory training (verbal and visuospatial working memory and storage tasks: 35–45 min/day, 5 days/week, 5 weeks)	Post-fitting
		Vitti et al. [61]	Internet-based auditory training (30 min/day, 5 days/week, 4 weeks)	Post-fitting
	Mobile	–	–	–
(4) General (tele-audiology)				
	Offline	–	–	–
	Internet	Eikelboom and Swanepoel [82]	Remote health and hearing services (e.g., video-conferencing, service delivery, e-mail use, Skype)	Pre-fitting, Fitting, Post-fitting
		Pearce et al. [83]	Remote hearing services (hearing assessment, HA fitting, hearing rehabilitation)	Pre-fitting, Fitting, Post-fitting
		Pross et al. [84]	Remote hearing services (video-conferencing, remote probe microphone and HA fitting)	Pre-fitting, Fitting, Post-fitting
	Mobile	–	–	–

*CVC* Consonant–Vowel–Consonant, *IOI-HA* International Outcome Inventory for Hearing Aids, *HA* hearing aid, *HADS* Hospital Anxiety and Depression Scale, *HHIE* Hearing Handicap Inventory for the Elderly, *HL* Hearing Loss, *PROM* patient-reported outcome measures, *PREM* patient-reported experience measures, *SADL* Satisfaction with Amplification in Daily Life

For counselling and patient education, eight records described provision of patient-oriented education and counselling, either *offline* (via DVD, PC, or telephone [22, 55–57]) or through the *Internet* [22, 54–56, 58–61]. For example, Ferguson et al. [22, 55, 56] demonstrated the potential benefits of a multimedia educational program based on “reusable learning objects” for HA users, delivered on PC, DVD, or through the Internet. These studies showed that eHealth can be used effectively to deliver learning and educational support as a supplement to clinical rehabilitation practice.

For the implementation of group discussions and forums, four records suggested *Internet*-*based* interaction with peers to support HA users [54, 58–60]. For example, Thorén et al. in their randomised controlled trial demonstrated that an online intervention program including self-studies, training and professional coaching, HAs, communication strategies, and online contact with peers can significantly reduce perceived hearing handicap in experienced HA patients [54]. For two out of three services in the area of education and information, evidence related to the pre-fitting phase was also found (Table 1). Specifically, two records were related to *Internet*-*based* delivery of HL and HA information to improve patients’ knowledge and help them developing awareness about their hearing problems and about the need for treatment and thee options available [50, 62]. Interestingly, Rothpletz et al. [62] showed that older adults who failed a hearing screening were likely to accept Internet delivery of hearing healthcare to begin to self-manage their hearing loss until they were ready to seek conventional clinical services. The study also showed that patients who underwent a brief training class reported increases in hearing healthcare knowledge and slight improvements in computer self-efficacy. Another study [56] investigated use of the Internet for counselling and patient education in the pre-fitting phase to support patients in their social and emotional needs, to modify attitudes and motivation, and to share information about choice of interventions. Interestingly, the study introduced a patient-centered intervention for persons with HL and their communication partners, based on the lived experiences of hearing disability with significant emphasis on ‘self-reflection’. The study failed to detect effects of intervention and no conclusions could be drawn due to limited sample size.

Of the five records related to the screening and assessment area [64–68], four were relevant to the pre-fitting phase (Table 2) as a means to detect HL. This was done through pure-tone audiometry on a portable system [64], through speech testing on the Internet or smartphone [65, 66], or through measures of hearing handicap/disability delivered online [67]. For example, Thorén et al. [67] compared administration online versus paper and pencil of four self-assessment questionnaires used in hearing research and clinic by using a cross-over design. The study provided evidence that participants’ scores remained consistent across administrations and formats for three of the four included questionnaires and observed a significant effect of format with small effect size for the fourth included questionnaire, with participants reporting more handicap when using the online format. In principle, online administration can save resources and lead to more complete answers when compared with conventional paper-and-pencil administration but as there may be slight differences in patient self-reported hearing handicap, the authors recommended that clinicians use consistent administration format across surveys and in follow up visits [67].

In three records, testing functionalities were also used to inform the following phase for appropriate fitting [65, 67] or sound compensation [66]. In the area of remote fitting, in particular, a study [65] showed that HA fittings based on an online speech perception test provided at least as much benefit in speech perception as fittings based on conventional methods—thus opening to use of remote testing as a means to reduce the costs and enable sustainable tele-audiology models. Evidence in the post-fitting phase was found only for some self-assessment measures, which were used as a means to monitor the patients in their daily life through Ecological Momentary Assessment [68]. The Authors investigated the impact of Ecological Momentary Assessment delivered through a personal digital assistant carried for 12 h a day during 2 weeks, and demonstrated the viability of the approach to address the experienced hearing problems, especially in challenging listening situations. Moreover, the study showed greater sense of awareness regarding HL and use of HA in study participants. This study is an interesting example of how mobile-based technology may enable individualized care as real-life outcome measures can be collected through the day and the within- and between-day variability in outcomes and associated factors can be addressed effectively [68].

In the hearing rehabilitation area, Table 1 shows that sound enhancement and HA control and fitting, as related to the technical aspects of amplification, were inherently related to the fitting phase [66, 69–74]. Smartphone-based sound enhancement was described by two records [66, 69]. These studies showed that sound enhancement can improve speech intelligibility and suggested that smartphone-based sound amplification, rather than being a substitute of HAs, can be used as a “starter” solution and provide temporary assistance to individuals with HL. Within the service HA control and fitting, this review showed examples of *Internet*-*based* remote control for real ear measurements [70] and HA fitting [23, 71]. For the former, minor differences between face-to-face and remote measurements were observed, within the inherent variability for probe microphone measurements [70]. For the latter, proven benefits and a high level of satisfaction were reported in participants who were fitted remotely [71]. Overall, these studies demonstrated the feasibility of delivering these services at a distance, with relevant implications for service delivery in underserved context or remote areas. This review also showed examples of *mobile*-*based* systems for HA control [72], self-fitting [73], or speech enhancement [74]. These studies showed that the use of mobile platforms can be useful to empower patients as the user can become part of the hearing device fine-tuning process [72]. Interestingly, one study demonstrated the need to provide knowledgeable support for HA self-fitting, possibly tailored according to previous HA experience and delivered by trained personnel rather than a fellow layperson [73]. Implications for designers and app developers were also highlighted as the cognitive, functional, and psychological aspects of the user experience should be taken into account in the development of mobile services for HA fitting and control [72].

The nine records related to auditory and cognitive training discussed this kind of intervention only in the post-fitting phase [55, 61, 75–81]—even though, in principle, auditory and cognitive training can also be provided before the fitting process. Across the nine records, different programs for auditory and working memory training were found (e.g., training with speech [55, 76–78], speech-in-noise [55, 76, 78], audio-visual stimuli [79], time-compressed stimuli [75, 78], visuospatial tasks [55, 76]), delivered either through PC or DVD [75–80] (*offline* platform) or through the *Internet* [55, 61, 76, 81]. These studies showed that offline platforms, in particular, have facilitated adaptive auditory training and the provision of communication strategies compared to face-to-face delivery [75, 78]. Olson et al. [78] concluded that many experienced HA users would have liked to have had auditory training before receiving HAs and that new users had increased benefits from training. The Authors pointed out that this might help in terms of lower return rates and improved cost-effectiveness. As for training programs delivered through the Internet, one study introduced audio-visual adaptive training delivered through games, puzzles, and videos for improving speech-in-noise understanding [81]. Another study introduced a program that targeted skills such as auditory closure, cognitive-linguistic, and communication strategies [61]. In general, use of eHealth can enable delivery of complex programs including combined auditory and cognitive training approaches, where cognitive enhancement is embedded within auditory tasks. These combined approaches are most likely to offer generalized benefits to the real-world listening abilities of adults with HL [76].

Finally, the three records in the general area of tele-audiology reported *Internet*-*based* remote activities related to all the phases in the patient journey, e.g. remote hearing assessment, fitting, counselling, rehabilitation [82–84]. Overall, these studies showed that tele-audiology is feasible for hearing assessment, HA fitting, and rehabilitative counseling. It can be as effective as in person audiology care and it may improve access especially in remote areas while preserving patients’ satisfaction [83, 84]. However, a recent international survey showed that despite audiologists have positive attitudes toward telehealth and associated technology, they reported limited clinical adoption of tele-audiology [82].

## Discussion

This state-of-the-art review identified 34 empirical studies related to eHealth and HA rehabilitation in adults and showed a varied picture of the several eHealth services that were introduced in the past decade to support patients throughout their journey. The review highlighted a growing interest in eHealth research in audiology throughout the years, as revealed by an increasing number of included records over the search period, from 2 in years 2009–2010 up to 17 in 2015–2016 (Fig. 1).

### *Internet*-*based* platforms

More than half of the included records (i.e., 21 out of 34) described services delivered over *Internet*-*based* platforms and the use of these platforms showed a stable trend throughout the years (Fig. 2). In general, eHealth services over the Internet have increased in popularity [85] together with the growing rate of internet usage, with 46.4% of the world population having internet access [86]. Not surprisingly, there is also a high prevalence of internet use among people with hearing problems, who have been shown to use the internet on average to the same extent as people with normal hearing [87]. Interestingly, elderly people with HL use the internet more than those with normal hearing [88, 89]. Documented benefits of using the Internet in HHC include, for example: dissemination and exchange of information on hearing and post-fitting care [8, 54, 60, 81], promotion of virtual communities [54, 62, 90], and digitally enabled audiology through cloud computing [11]. Internet-based services were used to support and complement rehabilitation in a typical audiological clinic, showing benefits in terms of increased participation, eased data collection, reduced time and cost [91], and improved patient outcomes [54, 60]. Examples of *Internet*-*based* services found in this review were, e.g.: patient portals [54, 62] or web-based group discussions and forums [54, 58, 60], remote control of clinical equipment for hearing testing and HA fitting [70, 71, 82–84], audio- and video-conferencing for tele-consultation and tele-audiology [82–84], or web-portals for online rehabilitation [22, 55, 56, 58, 61, 76, 81].

### *Offline* platforms

About one-third (i.e., 11 out of 34) of the included records introduced services over *offline* platforms (including use of telephone, DVDs, or PC-based applications). The introduction of PC-based applications has represented a significant step ahead in audiology because, compared to conventional face-to-face encounters, it can facilitate the implementation of adaptive exercises for auditory and cognitive training and it enables the provision of personalized communication strategies and tailored education to patients [75, 78, 92]. The use of *offline* platforms (especially, DVDs and PC) is common in audiology and has grown throughout the years, as reflected in the trends shown in Fig. 2. This can be due to full maturity of technology and large clinical evidence as well as, compared to Internet-based or mobile-based services, ease of use under less than ideal conditions (poor connectivity, underserved settings, or low technical skills). Some of the *offline* services highlighted by this review included, e.g., telephone-based consultations as a complement to a home-based educational program for HAs users [57], DVD- and PC-based programs for patient education, auditory rehabilitation, auditory and working memory training [22, 55–57, 75–80]. It is well known that the availability of Internet-based and mobile-based solutions has grown in the recent past and these platforms hold great promise for improving access to care and cost-efficiency. However, the advent of services over Internet-based and mobile-based platforms has not undermined the widespread use of more conventional DVD- and PC-based eHealth methods. This might be related to the fact that progress from innovation to clinical research and to clinical implementation typically takes several years. Several studies converged around an average time lag of 17 years in the development of health intervention (reviewed by [93]) but the process of getting evidence base into practice is complex and the time lag depends on many factors (e.g., domain, type of intervention, target population, and socioeconomic factors). Technology usage lags vary across countries, being highly correlated with inequalities in per-capita income [94]. In addition, differences are also observed across high-income countries. For example, differences in Internet use have been documented across Europe. Interestingly, one of the studies included in this review suggested that delivery of educational services using the Internet might be a barrier to effective service delivery for many first-time HAs users [22], and that this might be related to country-specific and socioeconomic factors. For example, Internet use in a large random sample of 65- to 74-years old was found to be lower in England [22, 89] than in Sweden [88]. Moreover, on a more general ground, use of Internet for service delivery can be effective to the extent that individuals are in a position to use it well. Therefore, the so-called “digital divide” along with demographic and socio-economic inequalities may create a gap between users and non-users [95]. Demographic and socio-economic inequalities among different population groups (e.g., related to ethnicity, socio-economic status, age and gender, literacy, health literacy, eHealth literacy, and access/affordability of technology) can lead to differences among individuals in terms of skills and ability to use technology effectively and this, in turn, can lead to (or exacerbate) health inequalities [95, 96]. So, efforts still need to be done to bring technology closer to those with reduced digital capabilities and health literacy so to enable any individual along the socio-economic scale to use eHealth technology effectively.

### *Mobile*-*based* platforms

In the very recent years, mHealth has been suggested as an accessible and ubiquitous means to bring healthcare closer to people in disadvantaged settings [8–10, 97]. In this review, we found interesting evidence about some services and functions that can be delivered over *mobile*-*based* platforms. Specifically, by using Personal Digital Assistants for patient monitoring [68], smartphones or tablets for hearing testing, personalized sound enhancement, or self-fitting [66, 69, 73]. The portable and instant nature of mobile devices and applications can enable on-demand information and data sharing, and open up for an abundance of personalized mHealth solutions connecting different actors such as professionals, patients, families and significant others. Apps are frequently described as appealing to the tech-savvy generation of baby-boomers and could, together with online home-based rehabilitation, supplement the professional toolbox and enable patient-centred healthcare [5, 7, 21]. At the same time, the issues and challenges of mHealth in HHC are still a matter of debate and the clinical adoption of mobile solutions is rather limited [5]. This may be due to several reasons. Possible issues of security, data protection, data use and reuse have been reported for mHealth apps, along with perceived risks related to safety and misuse, poor regulation and lack of standards for app validation, effectiveness, and quality [98–101]. A recent systematic review highlighted the need to raise the quality of mHealth intervention studies and to address the issues related to app use [102].

Interestingly, some services and functions can be delivered more easily through mobile technology, e.g., Ecological Momentary Assessment, sound enhancement, and hearing testing. Ecological Momentary Assessment (i.e. outcomes/experience sampling with field or real-time records of behaviours and situations) can be used to monitor patients through patient-reported outcome and experience measures (PROM/PREM) while in their own environment [68]. Sound enhancement, i.e. sound amplification and spectral shaping has been initially delivered by means of personal sound amplification products, i.e. over-the-counter personal devices not specifically labeled for hearing loss treatment [103, 104]. The advent of smartphones and mobile apps has paved the way to the development of a variety of apps for improved hearing [21]. These apps can either provide a list of preset configurations that the user can select based on the changing preferences in the different environments, or deliver an amplification profile that is computed from the person’s audiogram, as estimated by the same app through a hearing testing module [21, 69, 105].

Another promising area for the use of mobile technology and apps in HHC is the area of hearing testing. Although smartphone-based hearing testing presents several issues, especially lack of validation [3] and calibration of output levels [6, 44, 106–111], nevertheless it has important potential implications. Apps for hearing testing and screening are easy to use and can help citizens and patients to gain improved awareness of their hearing problems. Moreover, these apps frequently provide relevant information about hearing, HL and rehabilitation options so they can be helpful to improve knowledge and motivation and leverage patients’ readiness to seek help [3, 5, 6]. Effective use of mobile services for hearing screening and testing can potentially result in earlier detection and diagnosis, and improve help-seeking [6, 7]. The area of validation and calibration of hearing testing services delivered through apps is an important subject of research. Significant efforts have been made recently to try to address—and improve—the reliability and accuracy of mobile solutions for hearing assessment in non-clinical settings such as, for example, noisy environments [106, 107], primary health-care clinics [44], or underserved contexts [108, 109]. Some recent studies have investigated the reliability of using consumer technology (commercially available transducers) for hearing testing through smartphones [110] and suggested novel procedures for calibration that might be feasible on a mobile device (e.g., biological calibration of reference sound levels in relation to the normal-hearing thresholds of the participants [111]).

Overall, our review showed that the clinical penetration of mobile-based platforms in HHC is still limited. We found only one record related to smartphone-based hearing testing for sound compensation, one related to Ecological Momentary Assessment, and one related to sound enhancement. However, the reliability of mobile technology is projected to improve in the near future thanks to developments in technology and clinical research, so there might be an evolution of audiology-related mHealth solutions in the next years.

### eHealth services across the phases of the patient journey

The distribution of eHealth services across the included records (Fig. 3) showed that, overall, most of the eHealth services documented by this review were related to the service areas of education and information (42.5%) and hearing rehabilitation (40.4%), whereas 10.7% of the eHealth services were related to screening and assessment and the remaining 6.4% were related to tele-audiology in general across all the service areas.

A closer look at these data reveals that the three services most frequently reported by the assessed literature were: counselling and patient education (27.7%), auditory and cognitive training (23.4%), and HA control and fitting (12.8%). Interestingly, these three services constitute the core elements of a comprehensive intervention for adults with HL whereby HA fitting can be complemented with counselling and with tailored educational programs as well as by auditory training for overall better benefit and improved patient outcomes [75, 78, 92]. In fact, knowledge of HL and of communication strategies and auditory training increase social interaction [112], reduce hearing disability [54, 57, 60], and improve speech-in-noise recognition [78, 81]. Even simple strategies of audiological counselling such as, e.g., daily emails between patients and audiologists [113], can be powerful communication enablers as they can help exploring and understanding day-to-day experiences and facilitate more timely responses than face-to-face visits. A not negligible portion of studies were related to HL and HA information (6.4%) and group discussions and forums (8.51%) for patients, further documenting the importance of providing patients not only with amplification technology but also with relevant information about their hearing problems and the rehabilitation process as well as with platforms to interact with peers. The educational component of audiological intervention can be key to improve patient knowledge and awareness, increase motivation, and promote positive attitudes towards self-management and behaviour change [114, 115]. For patient education to achieve the expected benefits and act as a real facilitator of patient outcomes and satisfaction, effective delivery of information and educational materials is necessary. Research in this area is ongoing, especially to address the issues of readability and usability of health-related information. Recent studies investigated the quality and readability of internet information for adults with HL and demonstrated that people require between 9 and 14 years of education to be able to understand the online information currently available [116, 117] so low readability can be a barrier [118], especially for older people [119] and disadvantaged socioeconomic groups. The health- and eHealth-literacy of the target group are central to the uptake, adherence, and benefits of eHealth interventions. Evaluating the eHealth literacy of the target group, with one of the several validated instruments already available (e.g., [120]), and developing eHealth services accordingly is central for user involvement and empowerment. In addition to complex language, other barriers for implementing online information can be outdated websites [121], disabilities preventing patients to understand the online information such as visual or cognitive impairment [88], difficulties in navigation and low computer skills [119, 122]. For example, Eikelboom and Atlas [123] showed that patient involvement in eHealth was highly facilitated by previous awareness and knowledge of eHealth services and technology so user involvement by design should be considered in order to develop useful, effective solutions for patient education. As early as 2005, Wyatt and Sullivan pointed out that most eHealth solutions are developed from the basis of technological opportunity rather than on the user needs and expectations [124]. Frequently, novel solutions are tested in pilot studies to demonstrate technical feasibility but it takes much more effort to bring them in the clinic for real benefit. Participatory design can be key to this process [124, 125]. It is proved that patients can contribute to original innovations of health technology [126, 127].

### Limitations and future research

This review has some limitations. First, the search terms and inclusion/exclusion criteria used were broad, providing a comprehensive and descriptive review of the newest activities, efforts, and concerns in the context of eHealth and HAs, but also making specific conclusions more difficult to achieve. Second, this review only included records written in English language so relevant literature written in other languages might have been missed. Similarly, some relevant records might have been missed due to some excluded terms in the search string. For example, the term “sensorineural” was excluded as it is typically related to studies in the area of hearing pathology and mechanisms as well as in the area of infant hearing. Similarly, the term “implant” was excluded as we focused solely on HAs. Excluding these and other terms might have missed some relevant hits. Moreover, although the four databases we used gave ample coverage of the literature in the field, other ample databases were not considered (e.g., EMBASE) so it is possible that relevant studies were missed. Searching the reference lists of relevant records may have, at least in part, counterbalanced these effects. Finally, we chose to include only published research indexed on the four literature databases here used, but it is possible that searching on clinical trials databases (e.g., the ISRCTN Registry, ClinicalTrials.gov, or the WHO International Clinical Trials Registry Platform) would have provided additional results, especially about the latest completed and ongoing studies.

It is clear that eHealth shows promise in the field of HHC and, especially, in the context of HA rehabilitation in adults. Patient-centred care through self-management, education, self-testing and personalized rehabilitation, can lead to improved outcomes and higher satisfaction. There is currently a gap between the promises of eHealth and the available evidence documenting the benefits. To successfully inform and influence eHealth policies, the evidence must be of high quality. This systematic review showed that the average quality score of the 34 included records was about half of the maximum possible score. External validity and power were the two lowest scored quality domains. Poor external validity makes inference to the target population (e.g. HA users in general) difficult. Insufficient statistical power makes results difficult to generalize, as the study could fail to detect an effect, even though the effect occurs. Underpowered studies with poor external validity are a barrier for successful implementation of eHealth within audiology, as they cannot support effective evidence-based policy decisions regarding eHealth implementation. It would be important to conduct randomised and blinded studies combined with a priori power calculations to guide sample size decisions. Moreover, to take full advantage of the potential of eHealth in the context of HA rehabilitation as well as in hearing healthcare in general, it will be important to gain a deeper insight into the drivers and barriers to implementation as well as into the facilitators to improved outcomes. A systematic analysis of these elements would be essential to inform future research towards the development of eHealth solutions that leverage on the drivers and facilitators, try to limit the barriers to effective implementation, and enable improved strategies for service delivery for the different target user groups.

## Conclusions

Overall, this review showed that the field of eHealth in the context of HA rehabilitation in adults has grown in the past decade, with a large variety of services available throughout the phases of the patient journey, and with ample use of the *offline* and *Internet*-*based* platforms and a large room for improvement—and significant potential benefits—of *mobile*-*based* solutions. Large availability of eHealth services supporting self-help and a more personalized healthcare experience might fill a gap in conventional audiological services and meet patient expectations better through the use of pervasive technologies. The area of eHealth in audiology is growing and there is still need for research to further increase its penetration and efficacy in clinical practice. Research is needed both in terms of developments in technology along with technical and clinical validation as well as in terms of optimization of strategies for service delivery. Future research of high methodological quality, especially in terms of external validity and statistical power, will benefit effective evidence-based policy decisions on implementation of eHealth in audiology and related fields.

## Additional files

**Additional file 1.** Text giving the full search strings used for the four databases: CINAHL, PubMed, Scopus, and Web of Science.

**Additional file 2.** Assessment of study quality: methodology and results.

## Abbreviations

DVD: digital video disk
HA: hearing aid
HHC: hearing healthcare
HL: hearing loss
PC: personal computer
PICo: Population, Interest, and Context
PREM: patient-reported experience measures
PROM: patient-reported outcome measures
