# Supplementary material for: eHealth and the hearing aid adult patient journey: a state-of-the-art review
Source: Biomed Eng Online. 2018 Jul 31;17:101. doi: 10.1186/s12938-018-0531-3 (PMC6069792; doi:10.1186/s12938-018-0531-3)
Supplement: Supplementary file 1 — Additional file 1. Text giving the full search strings used for the four databases: CINAHL, PubMed, Scopus, and Web of Science. [file 12938_2018_531_MOESM1_ESM.docx]

**Additional file 1**

The full search strings used for the four databases.

**CINAHL**

TX ( ("hearing aid*" OR "sensory aid*" OR "hearing instrument*" OR "hearing loss" OR "hearing-related" OR "hearing healthcare") ) AND TX ( ("Tele-Health" OR "tele*medicine" OR "tele*audiology" OR "Tele*hearing" OR "eHealth" OR "Tele*practice" OR "remote consult*" OR "remote fit*" OR "remote" OR "mobile Health" OR "mhealth" OR "internet" OR "face-to face" OR "computer aided" OR "computer based" OR "online" ) ) NOT TX ( (( "cochlear implant*" OR "children*" OR "babies*" OR "hearing screening*" OR "implant*" OR "sensorineural" OR "visual*" OR "FM" OR "noise" OR "HIV" OR "CLS" OR "ELS" OR "SINS" OR "otitis media" OR "gene" OR "sens*" OR "congenital") Timespan 2007-2017 and English language

**PubMed:**

(("hearing aids"[MeSH Terms] OR ("hearing"[All Fields] AND "aids"[All Fields]) OR "hearing aids"[All Fields] OR ("hearing"[All Fields] AND "aid"[All Fields]) OR "hearing aid"[All Fields]) OR "sensory aid"[All Fields] OR "hearing instrument"[All Fields] OR "hearing loss"[All Fields] OR "hearing-related"[All Fields] AND (("internet"[MeSH Terms] OR "internet"[All Fields]) OR ("telemedicine"[MeSH Terms] OR "telemedicine"[All Fields] OR "ehealth"[All Fields]) OR ("telemedicine"[MeSH Terms] OR "telemedicine"[All Fields] OR "mhealth"[All Fields]) OR mobile[All Fields] OR "remote*"[All Fields] OR "tele-audiology"[All Fields] OR online[All Fields] OR "face-to face"[All Fields] OR "tele*health"[All Fields] OR (tele[All Fields] AND ("hearing"[MeSH Terms] OR "hearing"[All Fields])) OR "telemedicine"[All Fields] OR telepractice[All Fields] OR "Comput"[All Fields] OR "PC"[All Fields] OR "Computer*assist*"[All Fields] OR "Computer*aided"[All Fields] OR "Computer*based"[All Fields] OR "consulting"[All Fields] OR "fitt*"[All Fields]) NOT ("children"[MeSH Terms] OR "babies"[MeSH Terms] OR "cochlear implants"[MeSH Terms] OR ("cochlear"[All Fields] AND "implants"[All Fields]) OR "cochlear implants"[All Fields] OR ("cochlear"[All Fields] AND "implant"[All Fields]) OR "cochlear implant"[All Fields] OR "cochlear implantation"[MeSH Terms] OR ("cochlear"[All Fields] AND "implantation"[All Fields]) OR "cochlear implantation"[All Fields] OR ("cochlear"[All Fields] AND "implant"[All Fields])) NOT "(FM)"[All Fields] NOT "noise"[All Fields] NOT "HIV"[All Fields] NOT "CLS"[All Fields] NOT "ELS"[All Fields] NOT (visual[All Fields] OR visual'[All Fields] OR visual'no[All Fields] OR visual'noi[All Fields] OR visual3d[All Fields] OR visual3dx[All Fields] OR visuala[All Fields] OR visualable[All Fields] OR visualacuity[All Fields] OR visualanalogue[All Fields] OR visualant[All Fields] OR visualant'[All Fields] OR visualants[All Fields] OR visualase[All Fields] OR visualattention[All Fields] OR visualazation[All Fields] OR visualbalan[All Fields] OR visualbasic[All Fields] OR visualbyte[All Fields] OR visualcapacities[All Fields] OR visualcna[All Fields] OR visualcockpit[All Fields] OR visualcognition[All Fields] OR visualcontact[All Fields] OR visualcor[All Fields] OR visualcxtfit[All Fields] OR visualdecisionlinc[All Fields] OR visualdecisionlinc'[All Fields] OR visualdent[All Fields] OR visualdetection[All Fields] OR visualdmdx[All Fields] OR visualdx[All Fields] OR visuale[All Fields] OR visualed[All Fields] OR visualergonomics[All Fields] OR visuales[All Fields] OR visualeyes[All Fields] OR visualfield[All Fields] OR visualfields[All Fields] OR visualforensic[All Fields] OR visualfunction[All Fields] OR visualgenedeveloper[All Fields] OR visualgenomics[All Fields] OR visualiation[All Fields] OR visualidade[All Fields] OR visualied[All Fields] OR visualing[All Fields] OR visualis[All Fields] OR visualisable[All Fields] OR visualisant[All Fields] OR visualisar[All Fields] OR visualisatie[All Fields] OR visualisation[All Fields] OR visualisation'[All Fields] OR visualisations[All Fields] OR visualisatrice[All Fields] OR visualise[All Fields] OR visualised[All Fields] OR visualised'[All Fields] OR visualisee[All Fields] OR visualisees[All Fields] OR visualiser[All Fields] OR visualiser'[All Fields] OR visualiseras[All Fields] OR visualiseren[All Fields] OR visualiseret[All Fields] OR visualisering[All Fields] OR visualises[All Fields] OR visualisieren[All Fields] OR visualisierte[All Fields] OR visualisierter[All Fields] OR visualisierung[All Fields] OR visualisierungen[All Fields] OR visualisierungsalgorithmen[All Fields] OR visualisierungsform[All Fields] OR visualisierungsinstitut[All Fields] OR visualisierungsmodalitaten[All Fields] OR visualisierungssoftware[All Fields] OR visualisierungssystem[All Fields] OR visualisierungstechnik[All Fields] OR visualisierungstherapie[All Fields] OR visualisierungsverfahren[All Fields] OR visualising[All Fields] OR visualising'[All Fields] OR visualism[All Fields] OR visualisointi[All Fields] OR visualist[All Fields] OR visualistics[All Fields] OR visuality[All Fields] OR visualitzation[All Fields] OR ("radiography, dental, digital"[MeSH Terms] OR ("radiography"[All Fields] AND "dental"[All Fields] AND "digital"[All Fields]) OR "digital dental radiography"[All Fields] OR "visualix"[All Fields]) OR visualiza[All Fields] OR visualizability[All Fields] OR visualizable[All Fields] OR visualizables[All Fields] OR visualizacao[All Fields] OR visualizaci'on[All Fields] OR visualizacion[All Fields] OR visualizacoes[All Fields] OR visualizada[All Fields] OR visualizado[All Fields] OR visualizaion[All Fields] OR visualizaiton[All Fields] OR visualizando[All Fields] OR visualizar[All Fields] OR visualizaron[All Fields] OR visualizated[All Fields] OR visualizating[All Fields] OR visualization[All Fields] OR visualization'[All Fields] OR visualization's[All Fields] OR visualizational[All Fields] OR visualizations[All Fields] OR visualizations'[All Fields] OR visualizazione[All Fields] OR visualize[All Fields] OR visualize'[All Fields] OR visualized[All Fields] OR visualized'[All Fields] OR visualizedly[All Fields] OR visualizenetwork[All Fields] OR visualizer[All Fields] OR visualizer'[All Fields] OR visualizer's[All Fields] OR visualizers[All Fields] OR visualizers'[All Fields] OR visualizes[All Fields] OR visualizethe[All Fields] OR visualizible[All Fields] OR visualizing[All Fields] OR visualizsation[All Fields] OR visualiztion[All Fields] OR visualizzabili[All Fields] OR visualizzare[All Fields] OR visualizzarsi[All Fields] OR visualizzata[All Fields] OR visualizzatore[All Fields] OR visualizzazione[All Fields] OR visuall[All Fields] OR visuallacoustically[All Fields] OR visuallanguagelab[All Fields] OR visuallink[All Fields] OR visually[All Fields] OR visuallyand[All Fields] OR visuallyimpaired[All Fields] OR visuallzed[All Fields] OR visualmantis[All Fields] OR visualmente[All Fields] OR visualmining[All Fields] OR visualminteq[All Fields] OR visualmotor[All Fields] OR visualmotricity[All Fields] OR visualnastran[All Fields] OR visualness[All Fields] OR visualnet[All Fields] OR visualni[All Fields] OR visualnich[All Fields] OR visualnow[All Fields] OR visualoculomotor[All Fields] OR visualorthographic[All Fields] OR visualosonics[All Fields] OR visualpath[All Fields] OR visualpathway[All Fields] OR visualperceptual[All Fields] OR visualprot[All Fields] OR visualrank[All Fields] OR visualrecognition[All Fields] OR visualrecovery[All Fields] OR visualrepbase[All Fields] OR visualresults[All Fields] OR visualrtc[All Fields] OR visuals[All Fields] OR visualsample[All Fields] OR visualsensory[All Fields] OR visualsonic[All Fields] OR visualsonics[All Fields] OR visualspatial[All Fields] OR visualstents[All Fields] OR visualstruct[All Fields] OR visualstudio[All Fields] OR visualsurround[All Fields] OR visualsynchrony[All Fields] OR visualsystem[All Fields] OR visualtactile[All Fields] OR visualtapas[All Fields] OR visualtapas'[All Fields] OR visualte[All Fields] OR visualuized[All Fields] OR visualuzed[All Fields] OR visualvestibular[All Fields] OR visualy[All Fields] OR visualzing[All Fields] OR visualzzazione[All Fields]) NOT "otitis media"[All Fields] NOT "gene"[All Fields] NOT "sensorineural"[All Fields]) AND ("2007/01/01"[PDAT] : "2017/12/31"[PDAT])

**Scopus:**

TITLE-ABS-KEY ("hearing aid*" OR "sensory aid*" OR "hearing instrument*" OR "hearing loss" OR "hearing-related" OR "hearing healthcare") AND TITLE-ABS-KEY ( "Tele-Health" OR "tele*medicine" OR "tele*audiology" OR "Tele*hearing" OR "eHealth" OR "Tele*practice" OR "remote consult*" OR "remote fit*" OR "mobile Health" OR "mhealth" OR "internet" OR "face-to face" OR "computer aided" OR "computer based" OR "online" ) AND NOT ALL ( "cochlear implant*" OR "implant*" OR "sensorineural" OR "visual*" OR "FM" OR "noise" OR "HIV" OR "CLS" OR "ELS" OR "SINS" OR "otitis media" OR "gene" OR "sens*" OR "congenital" OR "child*" OR "bab*") AND PUBYEAR > 2006 AND LANGUAGE ( english ) AND ( LIMIT-TO(LANGUAGE,"English" ) ) AND ( EXCLUDE(DOCTYPE,"bk" ) ) AND ( EXCLUDE(SRCTYPE,"k" ) OR EXCLUDE(SRCTYPE,"b" ) )

**Web of Science:**

(TS=("hearing aid*" OR "sensory aid*" OR "hearing instrument*" OR "hearing loss" OR "hearing-related" OR "hearing healthcare") AND TS=("Tele*Health" OR "tele*medicine" OR "tele*audiology" OR "Tele*hearing" OR "eHealth" OR "Tele*practice" OR "remote consult*" OR "remote fit*" OR "mobile Health" OR "mhealth" OR "internet*" OR "face-to face" OR "computer assist*" OR "computer aided" OR "computer based" OR "online") NOT TS=("cochlear implant*" OR "implant*" OR "sensorineural" OR "visual*" OR "FM" OR "noise" OR "HIV" OR "CLS" OR "ELS" OR "otitis media" OR "gene" OR "children*" OR "babies*" OR "baby*")) *AND* **LANGUAGE:** (English)

Indexes=SCI-EXPANDED, SSCI, A&HCI, CPCI-S, CPCI-SSH Timespan=2007-2017
