# Supplementary material for: eHealth and the hearing aid adult patient journey: a state-of-the-art review
Source: Biomed Eng Online. 2018 Jul 31;17:101. doi: 10.1186/s12938-018-0531-3 (PMC6069792; doi:10.1186/s12938-018-0531-3)
Supplement: Supplementary file 2 — Additional file 2. Assessment of study quality: methodology and results. [file 12938_2018_531_MOESM2_ESM.docx]

**Additional file 2**

**Methodological quality assessment: Downs and Black^[[1]](#footnote-1)^ scale.**

| **Domain** | **Item** | **Scores** |
| --- | --- | --- |
| **Reporting** | **1**. Is the hypothesis/aim/objective of the study clearly described? | 1=Yes; 0=No |
|  | **2**. Are the main outcomes to be measured clearly described in the Introduction or Methods section? | 1=Yes; 0=No |
|  | **3**. Are the characteristics of the patients included clearly described? | 1=Yes; 0=No |
|  | **4**. Are the interventions of interest clearly described? | 1=Yes; 0=No |
|  | **5**. Are the distributions of principal confounders in each group of subjects to be compared clearly described? | 2=Yes 1=Partly; 0=No |
|  | **6**. Are the main findings of the study clearly described? | 1=Yes; 0=No |
|  | **7**. Does the study provide estimates of the random variability in the data for the main outcomes? | 1=Yes; 0=No |
|  | **8**. Have all important adverse events that may be consequence of the intervention been reported? | 1=Yes; 0=No |
|  | **9**. Have the characteristics of lost to follow-up been described? | 1=Yes; 0=No |
|  | **10**. Have actual probability values been reported (e.g. 0.035 rather than <0.05) for the main outcomes except where p <0.001? | 1=Yes; 0=No |
| **External validity** | **11**. Were the subjects asked to participate in the study representative of the entire population from which they were recruited? | 1=Yes; 0=No/Unable to determine |
|  | **12**. Were those subjects prepared to participate representative of the entire population from which they were recruited? | 1=Yes; 0=No 0=Unable to determine |
|  | **13**. Were the staff, places, and facilities representative of the treatment the majority of patients receive? | 1=Yes; 0=No 0=Unable to determine |
| **Internal validity and bias** | **14**. Was an attempt made to blind study subjects to the intervention they have received? | 1=Yes; 0=No 0=Unable to determine |
|  | **15**. Was an attempt made to blind those measuring the main outcomes of the intervention? | 1=Yes; 0=No 0=Unable to determine |
|  | **16**. If any of the results of the study were based on “data dredging”, was this made clear? | 1=Yes; 0=No 0=Unable to determine |
|  | **17**. In trials and cohort studies, do the analyses adjust for different lengths of follow-up of patients, or in case-control studies, is the time period between the intervention and outcome the same for cases and controls? | 1=Yes; 0=No 0=Unable to determine |
|  | **18**. Were the statistical tests used to assess the main outcomes appropriate? | 1=Yes; 0=No 0=Unable to determine |
|  | **19**. Was compliance with the intervention/s reliable? | 1=Yes; 0=No 0=Unable to determine |
|  | **20**. Were the main outcome measures used accurate (valid and reliable)? | 1=Yes; 0=No 0=Unable to determine |
| **Internal validity and confounding selection bias** | **21**. Were the patients in different intervention groups (trials and cohort studies) or were the cases and controls (case-control studies) recruited from the same population? | 1=Yes; 0=No 0=Unable to determine |
|  | **22**. Were study subjects in different intervention groups (trials and cohort studies) or were the cases and controls (case-control studies) recruited over the same period of time? | 1=Yes; 0=No 0=Unable to determine |
|  | **23**. Were study subjects randomised to intervention groups? | 1=Yes; 0=No 0=Unable to determine |
|  | **24**. Was the randomised intervention assignment concealed from both patients and health care staff until recruitment was complete and irrevocable? | 1=Yes; 0=No 0=Unable to determine |
|  | **25**. Was there adequate adjustment for confounding in the analyses from which the main findings were drawn? | 1=Yes; 0=No 0=Unable to determine |
|  | **26**. Were losses of patients to follow-up taken into account? | 1=Yes; 0=No 0=Unable to determine |
| **Power** (simplified version^[[2]](#footnote-2)^) | **27**. Does the study include a power calculation and use it accordingly? | 1=Yes  0=No |

**Methodological quality results: Sample size, study design, domain and overall quality of the 34 included records.**

| Record | Sample size | Study design^[[3]](#endnote-1)^ | **Quality scoring according to Downs and Black (1998)** | | | | | |
| --- | --- | --- | --- | --- | --- | --- | --- | --- |
|  |  |  | Reporting | External validity | Internal validity - bias | Internal validity – confounding | Power | Overall score |
| Abrams et al, 2015 | 29 | 3 | 8 | 1 | 5 | 3 | 0 | 16 |
| Aldaz et al, 2014 | 40 | 2 | 7 | 0 | 2 | 0 | 0 | 9 |
| Amlani et al, 2013 | 18 | 2 | 7 | 0 | 4 | 2 | 1 | 13 |
| Blamey, Blamey & Saunders, 2015 | 1409 | 2 | 4 | 1 | 5 | 1 | 0 | 10 |
| Chisolm et al, 2013 | 50 | 2 | 7 | 0 | 3 | 2 | 0 | 11 |
| Coco et al, 2015 | 63 | 2 | 5 | 1 | 2 | 0 | 0 | 8 |
| Convery et al, 2015 | 40 | 2 | 8 | 2 | 5 | 2 | 0 | 16 |
| Eikelboom & Swanepoel, 2016 | 269 | 2 | 4 | 0 | 4 | 2 | 0 | 9 |
| Ferguson & Henshaw, 2015a | 231 | 3 | 5 | 1 | 5 | 3 | 0 | 13 |
| Ferguson & Henshaw, 2015b | 131 | 3 | 6 | 1 | 5 | 3 | 0 | 14 |
| Ferguson et al, 2015a | 203 | 3 | 8 | 2 | 6 | 6 | 0 | 21 |
| Ferguson et al, 2015b | 203 | 3 | 8 | 2 | 5 | 6 | 0 | 20 |
| Ferrari & Bernandez-Braga, 2009 | 60 | 2 | 5 | 0 | 2 | 0 | 0 | 7 |
| Galvez et al, 2012 | 24 | 2 | 7 | 0 | 3 | 0 | 0 | 10 |
| Henshaw et al, 2015 | 44 | 3 | 6 | 2 | 5 | 4 | 0 | 16 |
| Lundberg et al, 2011 | 69 | 3 | 8 | 0 | 3 | 4 | 1 | 16 |
| Malmberg et al, 2017 | 74 | 3 | 9 | 2 | 6 | 5 | 1 | 22 |
| Manchaiah et al, 2014 | 80 | 3 | 10 | 3 | 6 | 6 | 1 | 25 |
| Olson et al, 2013 | 29 | 3 | 9 | 3 | 4 | 3 | 1 | 19 |
| Panahi et al, 2016 | 20 | 2 | 5 | 1 | 4 | 1 | 0 | 11 |
| Pearce et al, 2009 | 5 | 1 | 3 | 0 | 2 | 0 | 0 | 5 |
| Peddie and Kelly-Campbell, 2017 | 11 | 2 | 7 | 0 | 4 | 2 | 0 | 12 |
| Penteado et al, 2012 | 3 | 1 | 3 | 0 | 0 | 0 | 0 | 3 |
| Penteado et al, 2014 | 8 | 2 | 5 | 2 | 1 | 2 | 0 | 9 |
| Preminger & Rothpletz, 2016 | 163 | 2 | 4 | 1 | 3 | 1 | 0 | 8 |
| Pross et al, 2016 | 42697 | 2 | 4 | 2 | 5 | 2 | 1 | 13 |
| Rishiq et al, 2016 | 24 | 3 | 7 | 1 | 4 | 3 | 0 | 14 |
| Rothpletz et al, 2016 | 27 | 3 | 8 | 0 | 4 | 3 | 0 | 14 |
| Saunders et al, 2016 | 279 | 3 | 8 | 2 | 6 | 5 | 1 | 21 |
| Sohn et al, 2011 | 56 | 2 | 4 | 0 | 0 | 0 | 0 | 4 |
| Thoren et al, 2011 | 59 | 3 | 8 | 0 | 5 | 5 | 0 | 17 |
| Thoren et al, 2012 | 65 | 2 | 10 | 2 | 4 | 6 | 0 | 21 |
| Thoren et al, 2014 | 76 | 3 | 8 | 0 | 5 | 5 | 0 | 17 |
| Vitti et al, 2015 | 150 | 2 | 2 | 0 | 0 | 0 | 0 | 2 |

**Methodological quality results – overview.**

|  |  |  | **Study design** | | |
| --- | --- | --- | --- | --- | --- |
|  |  | **All records** | **RCTs** | **Cross-sectional /Cohort studies** | **Case studies** |
|  |  | (N = 34) | (N = 15) | (N = 17) | (N = 2) |
| **Overall** | Average | 13.1 (47%) | 17.7 (63%) | 10.2 (36%) | 4.0 (14%) |
|  | s.d. | 5.8 | 3.6 | 4.3 | 1.4 |
|  | Range | 2-25 | 13-25 | 2-21 | 3-5 |
| **Domains** | Reporting | 6.4 (58%) | 7.7 (70%) | 5.6 (51%) | 3.0 (27%) |
|  | External validity | 0.9 (30%) | 1.3 (43%) | 0.7 (23%) | 0.0 (0%) |
|  | Internal validity, bias | 3.7 (53%) | 4.9 (70%) | 3.0 (43%) | 1.0 (14%) |
|  | Internal validity, confounding | 2.6 (43%) | 4.3 (72%) | 1.3 (22%) | 0.0 (0%) |
|  | Power | 0.2 (21%) | 0.3 (33%) | 0.1 (12%) | 0.0 (0%) |

1. Downs SH, Black N. The feasibility of creating a checklist for the assessment of the methodological quality both of randomised and non-randomised studies of health care interventions. J Epidemiol Community Health. 1998;52:377-384. [↑](#footnote-ref-1)
2. O’Connor SR, Tully MA, Ryan B, Bradley JM, Baxter GD, McDonough SM. Failure of a numerical quality assessment scale to identify potential risk of bias in a systematic review: a comparison study. BMC Res Notes 2015;8:224. [↑](#footnote-ref-2)
3. 1= Case study, 2= Cross-sectional / Cohort study, 3= Randomized controlled trial [↑](#endnote-ref-1)
